# Clustering networked funded European research activities through rank-size laws

**DOI:** 10.1007/s10479-023-05321-6

**Published:** 2023-05-05

**Authors:** Roy Cerqueti, Antonio Iovanella, Raffaele Mattera

**Affiliations:** 1 Department of Social and Economic Sciences, Sapienza University of Rome, P.le A. Moro, 5, 00185 Rome, Italy; 2 GRANEM, Université d’Angers, Angers, France; 3 School of Economics, University of International Studies of Rome - UNINT, Via Cristoforo Colombo, 200, 00147 Rome, Italy

**Keywords:** Rank-size analysis, Complex networks, Cluster analysis, European research projects

## Abstract

This paper treats a well-established public evaluation problem, which is the analysis of the funded research projects. We specifically deal with the collection of the research actions funded by the European Union over the 7th Framework Programme for Research and Technological Development and Horizon 2020. The reference period is 2007–2020. The study is developed through three methodological steps. First, we consider the networked scientific institutions by stating a link between two organizations when they are partners in the same funded project. In doing so, we build yearly complex networks. We compute four nodal centrality measures with relevant, informative content for each of them. Second, we implement a rank-size procedure on each network and each centrality measure by testing four meaningful classes of parametric curves to fit the ranked data. At the end of such a step, we derive the best fit curve and the calibrated parameters. Third, we perform a clustering procedure based on the best-fit curves of the ranked data for identifying regularities and deviations among years of research and scientific institutions. The joint employment of the three methodological approaches allows a clear view of the research activity in Europe in recent years.

## Introduction

The funded joint projects can well describe the scientific interconnections among Research Institutions and Universities. Thus, one can observe an intuitive complex network structure of the research activity(Amoroso et al., 2018; Barber et al., 2006; Heller-Schuh et al., 2011; Lee et al., 2012; Schütz & Strohmaier, 2020).

In this context, the research actions funded by the European Union represent a relevant, high-quality instance for a clear view of such scientific interrelations and their evolution over time(Bastidon & Parent, 2022; Cerqueti et al., 2022; Cinelli et al., 2021).

One of the most relevant factors towards success in the development and implementation of projects and innovative initiatives is the presence of an innovative environment, characterized by a network of public and private actors and analyzed under different perspectives(Bogers et al., 2017); in particular, networking between research centers, industries and public institutions are often referred as the Triple Helix model(Etzkowitz & Leydesdorff, 1995), which finds application also for the European research funding schemes(Etzkowitz, 2002). Networks of public-private partnerships were analysed under different point of view, with an evaluation of their impact on rural areas(Esparcia, 2014), consortium characteristics(Wanzenböck et al., 2020), engagement of actors(Huggins et al., 2020), effectiveness (de Arroyabe et al., 2021) or on the scientific literature(Bergé et al., 2017).

This paper enters this theme. We build time-dependent networks associated with the European research projects generated by the data available at the official portal for European data regarding the 7th Framework Programme for Research and Technological Development (FP7) and the Horizon 2020 (H2020).

Each network corresponds to one year of research projects. The nodes are the participants in European projects, namely higher education institutions (HES), research organizations (REC), public bodies (PUB), private sector (PRC) or other participants (OTH). An edge connects them if they participated in the same project. The reference period is from 2007 to 2013 for FP7 and from 2014 to 2020 for H2020.

The final scope of the paper is to cluster years of networked research projects on the basis of two main criteria. On one side, we adopt some meaningful centrality measures of the networks, each of which has relevant informative content on the considered scientific interconnections. On the other side, we provide a rank-size analysis of such centrality measures to create a unified system from the granular data of the individual organizations. In doing so, we give a comparison among years of research/networks based on how research organizations form an overall system as interconnected entities, according to their nodal centrality measures. Specifically, we exploit the functional properties of the best-fit curves, that depend on the values of the calibrated parameters. As we will see below, such properties lead to an illustration of the way the nodes are related with the others in terms of their centrality measures. The clustering exercise illustrates similarities and discrepancies among the considered years of research on the ground of the involved networked research institutions. Therefore, such a statistical procedure gives a clear idea on the topological structure of the networks and, consequently, on the role of the institutions within the collections of research projects starting in a given year. More in detail and as we will see below, the clustering procedure allows us to detect similar hubs over the years–hence, lumping together years when institutions had analogous scientific connections with the others–or years with a similar set of institutions playing a leading role in connecting the others.

As intuition suggests, the reference methodological literature comprises three areas: complex network theory, rank-size analysis and cluster analysis.

Complex networks theory is an interdisciplinary field of studies aiming to understand the structure, development and dynamics of networks through different methods and tools attributed to several disciplines such as mathematics, statistics, physics, and computer science(Newman, 2018). In this respect, it can be considered as an abstraction of observable reality able to explain the performance of real systems since it correlates form with functions and structure with behaviours(Barabási, 2016; Lewis, 2009). Complex networks tools can be significant in revealing complex systems’ underlying structure and organization, which can be evaluated with quantitative measures. This explains the popularity of complex networks for modelling purposes in contexts like social network(Borgatti & Everett, 2006; Scott & Carrington, 2011), but also significantly in finance(Bastidon & Parent, 2022; Boginski et al., 2006; Cerqueti et al., 2021, 2022; Cinelli et al., 2021; Yan & Qi, 2021; Zhu et al., 2018), or healthcare and physiology (Butenko & Wilhelm, 2006; Vieira et al., 2010) and even for the understanding of the historical patterns (Pablo-Martí et al., 2021).

The rank-size analysis is a statistical methodology that allows deriving a unique system from disaggregated and properly ordered data. The starting point is a collection of some observations of a quantitative phenomenon – the size; then, such observations are ranked in decreasing order. The observation with the highest value has rank 1 and the one with the lowest value has a rank equal to the cardinality of the observed sample. In doing so, we obtain a descending scatter plot in the rank-size plan, which a decreasing curve can approximate through a best-fit procedure. The selection of the parametric family of curves and the value of the calibrated parameters provides information about the system’s structure described by the ranked data. In this, the optimal best-fit curve describes the properties and regularities of the observed sample as a unique set. There are important applications of rank-size analysis in the literature (see e.g., Ausloos, 2014; Cerqueti et al., 2022b; Ficcadenti & Cerqueti, 2017; Ficcadenti et al., 2019; Gabaix, 1999a, b; Vitanov & Ausloos, 2015). The most widely used parametric families of curves are the power law, the Zipf law (see Zipf , 1949) and the Zipf-Mandelbrot law (see Mandelbrot , 1953). Recently, (Ausloos & Cerqueti, 2016) introduced a so-called universal law, which allows us to capture system deviations at high and low ranks. In this work, we test the four aforementioned laws, obtaining that the universal law can suitably approximate the considered systems related to these years of research funding.

Finally, cluster analysis is an unsupervised learning task that aims to find groups of similar units. To this aim, defining a dissimilarity measure among the statistical units is crucial. In what follows, we aim to cluster complex networks – i.e. the years of networked research projects – through rank-size laws. In this way, the dissimilarities are determined on the basis of parameters characterizing the rank-size curves. We take inspiration from a strand of literature proposing clustering approaches of model-based type, where clusters are identified on the basis of parameters estimated from a statistical model. Examples are, among the others, the parameters of ARMA(Corduas & Piccolo, 2008) and GARCH(Caiado & Crato, 2010) processes or cepstral coefficients (D’Urso et al., 2020) for time series data, the regression coefficients for spatial data (Lee et al., 2020; Kopczewska, 2021; Kopczewska & Ćwiakowski, 2021) but also the parameters of probability distributions(Wang et al., 2011; D’Urso et al., 2017; Cerqueti et al., 2021, 2022a). In our setting, the model is the rank-size law. The rank-size curves’ parameters are used, for example, for clustering time series related to COVID-19 at a country level (see Cerqueti & Ficcadenti , 2022) and Italian soccer championships and teams (see Ficcadenti et al. , 2022). In what follows, we propose a novel rank-size approach for clustering complex networks in the context of research projects.

We take degree, betweenness, closeness and eigenvector as centrality measures. Such selected instruments capture different aspects of the considered networks, identifying those nodes that are best connected to others or have the most influence, indicating highly and tightly linked ones.

As already discussed above and for any nodal centrality measure, we rank the nodes in decreasing order so that rank 1 is associated with the largest value of the centrality measure while the highest rank is associated with the lowest value of the centrality measure. Then, we implement a best-fit procedure on the four parametric decreasing curves mentioned above – the power, the Zipf, the Zipf-Mandelbrot and the universal law – and identify the family leading to a statistically satisfactory data representation, along with the calibrated parameters. The clustering exercise and the interpretation of the calibrated parameters lead to the assessment of similarities or deviations in how scientific communities have conducted research over the years.

The proposed rank-size clustering procedure is based on two main steps. In the first step, we estimate the parameters characterizing the rank-size law through non-linear least squares regression. In the second step, considering a Euclidean distance among the estimated parameters, we use the fuzzy *k*-medoids clustering algorithm of Krishnapuram et al. (2001). First, we adopt a Partition Around Medoids (PAM) approach because it is more robust to the presence of outliers than other available alternatives, like the *k*-means. Second, we consider a fuzzy approach to account for the uncertainty in the clustering process. Indeed, fuzzy clustering allows a statistical unit to be allocated to more clusters with a membership degree representing the uncertainty related to its assignment. Results offer relevant insights into the scientific institutions and years of funded research.

To the best of our knowledge, this is the first time that the networks of the EU research funds are considered along with their centrality measures and clustered on the basis of a rank-size analysis.

The paper is structured as follows. Section 2 is devoted to developing the research network models, with a focus also on the considered data. Section 3 outlines the methodological techniques used for carrying out the study, with a proper distinction among complex network theory, rank-size analysis and clustering models. Section 4 collects the main results of the study, along with related comments. In the end, Section 5 provides some conclusions and lines for future research.

## The European collaborative research networks model

### Data setting and preprocessing

We use data provided by the European Commission regarding the 7th Framework Programme for Research and Technological Development (FP7) and the Horizon 2020 (H2020). Such initiatives are the most significant EU Research and Innovation programmes, and they have the active strategic objective of fostering scientific and technological development across Europe. FP7 was active from 2007 to 2013 with a total budget of over €50 billion^1^, while H2020 was active from 2014 to 2020 with a total budget of over €80 billion^2^.

Both FP7 and H2020 data come in the form of a table with columns listing project acronyms and respective participants, and their network properties have been a matter of recent investigations(Balland et al., 2019; de Arroyabe et al.,, 2021; Heller-Schuh et al., 2011). We extract from the tables the projects having the starting date within a given year. In so doing, we construct 14 networks of collaboration, one for each year: from 2007 to 2013 for FP7 and from 2014 to 2020 for H2020.

For each year, we build up a bipartite network $G(V_1, V_2, E)$ in which one partition ($V_1$) is made up of financed projects while the other ($V_2$) is made up of participants to such projects. A link in *E* between the partitions exists if an organization participated in a project. We then project the bipartite network onto the participants’ partitions (through an operation called one-mode projection(Newman, 2018)), thus obtaining another network $G'(V_2, E')$ in which two organizations in $V_2$ are connected if they participate in the same project in $V_1$.

**Table 1:** Main information about yearly networks of financed research projects

	Year	*n*	*m*	HES	OTH	PRC	PUB	REC
FP7	2007	452	4594	132	23	141	63	93
	2008	7750	136606	1175	408	4074	618	1315
	2009	6880	111865	1123	489	3320	523	1317
	2010	7806	119435	1186	610	4112	501	1312
	2011	8341	137220	1162	600	4452	535	1467
	2012	9188	128863	1119	614	5478	538	1334
	2013	9808	183728	1177	664	5804	588	1442
H2020	2014	5088	72990	854	458	2492	393	834
	2015	9625	149384	1205	1174	5108	810	1315
	2016	9081	157975	1273	1041	4718	790	1259
	2017	9263	156812	1250	1139	4841	795	1238
	2018	8417	160690	1195	1126	4297	676	1123
	2019	9145	169377	1246	1168	4641	800	1290
	2020	4133	67521	900	487	1724	299	723

Size (*n*), dimension (*m*) and network nodes’ types are reported for each network (Year). The types of organization are: higher education institution (*HES*), research organization (*REC*), public body (*PUB*), private sector (*PRC*) or other participants (*OTH*).

We repeat this procedure for all the 14 years. Thus we obtain 14 undirected networks, one for each year. The number of nodes *n* and links *m* for each projected network are reported in Table 1.

The same table also reports the number of nodes for each type of organization. Indeed, the original data reports information about the type of each organization, which can be: higher education institution (HES), research organization (REC), public body (PUB), private sector (PRC) or other participants (OTH). Table 2 shows the same information in percentage, and it is possible to see how the PRC constitutes the most prominent type, ranging from $31.2\%$ (2007) to $59.6\%$ (2012). However, if we rank organizations by their network degree *k*, i.e., their number of connections (see Table 6), we can note a different setting. In particular, Table 3, Table 4 and Table 5 show that organizations that are in the top ten rank according to their degree for at least one the observed years $2007, \ldots , 2020$. They are thirty different organizations and are limited to higher educational institutes (HES, 12 organizations), research organizations (REC, 15 organizations) and public bodies (PUB, 3 organizations).

**Table 2:** Percentage of nodes’ types for each network (Year)

	Year	$\%$HES	$\%$OTH	$\%$PRC	$\%$PUB	$\%$REC
FP7	2007	0.292	0.051	0.312	0.139	0.206
	2008	0.152	0.053	0.526	0.080	0.170
	2009	0.163	0.071	0.483	0.076	0.191
	2010	0.152	0.078	0.527	0.064	0.168
	2011	0.139	0.072	0.534	0.064	0.176
	2012	0.122	0.067	0.596	0.059	0.145
	2013	0.120	0.068	0.592	0.060	0.147
H2020	2014	0.168	0.090	0.490	0.077	0.164
	2015	0.125	0.122	0.531	0.084	0.137
	2016	0.140	0.115	0.520	0.087	0.139
	2017	0.135	0.123	0.523	0.086	0.134
	2018	0.142	0.134	0.511	0.080	0.133
	2019	0.136	0.128	0.507	0.087	0.141
	2020	0.218	0.118	0.417	0.072	0.175

Note that some rows do not sum to 1 since, for some organizations (the nodes), the type is not reported. The types of organization are: higher education institution (HES), research organization (REC), public body (PUB), private sector (PRC) or other participants (OTH).

**Table 3:** Organizations that are in the top ten rank according to their degree for at least one the observed years $2007, \cdots , 2020$ – main information

	Name	Type	Country
1	Aarhus Universitet	HES	DK
2	Agencia Estatal Consejo Superior Deinvestigaciones Cientificas	REC	ES
3	Bundesministerium fuer Bildung und Forschung	PUB	DE
4	Centre National de la Recherche Scientifique Cnrs	REC	FR
5	Commissariat à l’Energie Atomique et aux Énergies Alternatives	REC	FR
6	Consiglio Nazionale Delle Ricerche	REC	IT
7	Danmarks Tekniske Universitet	HES	DK
8	Deutsches Zentrum fuer Luft- und Raumfahrt	REC	DE
9	École Polytechnique Fédérale De Lausanne	HES	FR
10	Eidgenoessische Technische Hochschule Zuerich	HES	CH
11	Ethniko Kentro Erevnas Kai Technologikis Anaptyxis	REC	GR
12	Fraunhofer Gesellschaft zur Foerderung der Angewandten Forschung E.V.	REC	DE
13	Fundacion Tecnalia Research & Innovation	REC	SP
14	Imperial College Of Science Technology And Medicine	HES	GB
15	Institut National de la Santé et de la Recherche Médicale	HES	FR
16	Istituto Nazionale di Fisica Nucleare	REC	IT
17	Jrc - Joint Research Centre - European Commission	REC	EU
18	Katholieke Universiteit Leuven	HES	BE
19	Nederlandse Organisatie voor Toegepast Natuurwetenschappelijk Onderzoek Tno	REC	NL
20	Politecnico Di Milano	HES	IT
21	Science and Technology Facilities Council	REC	GB
22	Sofia University St Kliment Ohridski	HES	BG
23	Stichting Wageningen Research	REC	NL
24	Technische Universiteit Delft	HES	NL
25	Teknologian Tutkimuskeskus	REC	FI
26	The Icelandic Centre For Research	PUB	IS
27	The University Of Manchester	HES	GB
28	United Kingdom Research And Innovation	REC	GB
29	University College London	HES	GB
30	Vetenskapsradet - Swedish Research Council	PUB	SE

**Table 4:** Organizations that are in the top ten rank according to their degree for at least one the observed years $2007, \cdots , 2020$ – years 2007–2013

Name	2007	2008	2009	2010	2011	2012	2013
Aarhus Universitet							
Agencia Estatal Consejo Superior		6	6	5	8	7	4
Deinvestigaciones Cientificas							
Bundesministerium fuer Bildung und	10						
Forschung							
Centre National de la Recherche Sci-	1	2	1	2	2	2	2
entifique Cnrs							
Commissariat a‘ l’Energie Atomique et aux E’nergies Alternatives	6	3	5	3	3	4	3
Consiglio Nazionale Delle Ricerche	2	4	3	4	5	3	5
Danmarks Tekniske Universitet					10	5	7
Deutsches Zentrum fuer Luft- und	4		8				
Raumfahrt							
E’cole Polytechnique F’ed’erale De Lau-				10			
sanne							
Eidgenoessische Technische		7		8			
Hochschule Zuerich							
Ethniko Kentro Erevnas Kai Tech-							
nologikis Anaptyxis							
Fraunhofer Gesellschaft zur Fo-		1	2	1	1	1	1
erderung der Angewandten Forschung							
E.V.							
Fundacion Tecnalia Research & Inno-						9	9
vation							
Imperial College Of Science Technol-		9		6	9		
ogy And Medicine							
Institut National de la Sant’e et de la							
Recherche M’edicale							
Istituto Nazionale di Fisica Nucleare	7						
Jrc - Joint Research Centre - Euro-		10	7		7		
pean Commission							
Katholieke Universiteit Leuven			10	7			10
Nederlandse Organisatie voor			9	9	4	6	6
Toegepast Natuurwetenschappelijk							
Onderzoek Tno							
Politecnico Di Milano							
Science and Technology Facilities	5						
Council							
Sofia University St Kliment Ohridski	8						
Stichting Wageningen Research						10	
Technische Universiteit Delft							
Teknologian Tutkimuskeskus		5	4		6	8	8
The Icelandic Centre For Research	3						
The University Of Manchester		8					
United Kingdom Research And Inno-							
vation							
University College London							
Vetenskapsradet - Swedish Research	9						
Council

**Table 5:** Organizations that are in the top ten rank according to their degree for at least one the observed years $2007, \cdots , 2020$ – years 2014–2020

Name	2014	2015	2016	2017	2018	2019	2020
Aarhus Universitet				9			7
Agencia Estatal Consejo Superior	8	5	4	6	6	5	6
Deinvestigaciones Cientificas							
Bundesministerium fuer Bildung und							
Forschung							
Centre National de la Recherche Sci-	5	2	3	4	4	2	1
entifique Cnrs							
Commissariat a‘l’Energie Atomique et aux E’nergies Alternatives	3	3	5	2	3	3	4
Consiglio Nazionale Delle Ricerche	4	4	2	3	2	4	2
Danmarks Tekniske Universitet	9		6		8	9	5
Deutsches Zentrum fuer Luft- und				10		10	
Raumfahrt							
E’cole Polytechnique F’ed’erale De Lau-							
sanne							
Eidgenoessische Technische		7					
Hochschule Zuerich							
Ethniko Kentro Erevnas Kai Tech-						6	
nologikis Anaptyxis							
Fraunhofer Gesellschaft zur Fo-	1	1	1	1	1	1	3
erderung der Angewandten Forschung							
E.V.							
Fundacion Tecnalia Research & Inno-			8		5		8
vation							
Imperial College Of Science Technol-							
ogy And Medicine							
Institut National de la Sant’e et de la							10
Recherche M’edicale							
Istituto Nazionale di Fisica Nucleare							
Jrc - Joint Research Centre - Euro-	10						
pean Commission							
Katholieke Universiteit Leuven					7	7	
Nederlandse Organisatie voor	2	9		7			
Toegepast Natuurwetenschappelijk							
Onderzoek Tno							
Politecnico Di Milano				8			
Science and Technology Facilities							
Council							
Sofia University St Kliment Ohridski							
Stichting Wageningen Research							
Technische Universiteit Delft	6		7		10		
Teknologian Tutkimuskeskus	7	6	10	5	9	8	9
The Icelandic Centre For Research							
The University Of Manchester							
United Kingdom Research And Inno-		10					
vation							
University College London		8	9				
Vetenskapsradet - Swedish Research							
Council							

## Methodology

This section provides the methodological devices used for the analysis.

### Centrality measures

This paper considers some of the main centrality measures, which are commonly used in complex and social network analysis to assess the involvement of nodes in network(Borgatti & Everett, 2006; Scott & Carrington, 2011). Indeed, centrality measures represent the relative importance – in some sense – of a node within a network, with the assertion that the higher the centrality index of a node, the higher its perceived centrality in the graph.

Several centrality measures describe the node’s involvement, all of them with specific informative content. Hence, deciding which option to choose requires some consideration of the system under observation and the aspects to be highlighted. In other terms, the concept of centrality has an inherent ambiguity, and there is no point in including all measures in one method(Rowley, 1997).

For all the 14 networks, we consider: degree centrality *k*, closeness centrality $C_C$, betweenness centrality $C_B$ and eigenvector centrality $C_E$. A brief description of such measures with their informative content is reported in Table 6.

**Table 6:** A short glossary of centrality measures (adapted from Ferraro and Iovanella (2017)). Please refer to Scott and Carrington (2011) for a complete description and formulation

Measure	Definition	Meaning	Interpretation
Degree centrality (*k*)	The number of links incident upon a node, which can be interpreted as the neighborhood size of each member within the network.	This value highlights the immediate risk of a node catching whatever is flowing through the network. It quantifies how well it is connected to the other elements of the graph. The degree of centrality is an indicator of the spread of node connectivity along the graph and is a crucial gauge in defining the network organization.	It quantifies the number of scientific institutions linked with a given one through collaborations in the same research projects.
Closeness centrality ($C_C$)	The natural distance between all pairs of nodes are defined by the length of their shortest paths. Thus, the more central a node is, the lower its distance is from all other nodes.	This value measures how long it takes to spread information from a member to all others sequentially.	It measures the degrees of separation among the institutions in terms of scientific collaborations.
Betweenness centrality ($C_B$)	The number of times a node acts as a bridge along the shortest path between two other nodes.	This measure reveals the intermediary members that are essential for connecting different regions of the network.	It provides information on the role of a scientific institution in connecting the others through common research projects.
Eigenvector centrality ($C_E$)	The influence of a node in a network according to the number and the *quality* of its connections – where *high quality* means that the given node is connected with other well-connected nodes.	Indeed, a node with a smaller number of high-quality links has more power than one with a larger number of mediocre contacts.	It measures the number and the quality of the institutions linked to a given one in terms of their scientific connections through common research projects.

### Rank-size analysis

We rank the nodes according to the centrality measures described in Subsection 3.1 in descending order, so that the node with the highest value of the centrality measure is ranked to $r=1$. We implement a ranking exercise for the four centrality measures.

Let define the size as *y* and $f(r,\theta )$ the rank-size law with *r* the rank and $\theta$ be the vector including the rank-size parameters. We test the following four rank-size curves:

- Power law
  1 $$\begin{aligned} y= f(r, \theta ) = f(r, A, \alpha )=\frac{A}{r^\alpha }, \end{aligned}$$
  where *r* is the rank, while *A* and $\alpha$ are positive parameters to be calibrated.
- Zipf-Mandelbrot law
  2 $$\begin{aligned} y=f(r, \theta )= f(r, B, \lambda , \beta )=\frac{B}{(r+\lambda )^\beta }, \end{aligned}$$
  where *r* is the rank, while *B*, $\lambda$ and $\beta$ are positive parameters to be calibrated.
- Exponential law
  3 $$\begin{aligned} y=f(r, \theta )= f(r, C, \gamma ) = C\exp \left( -\gamma r\right) \end{aligned}$$
  where *y* is the size, *r* is the rank and $C, \gamma$ are positive parameters to be calibrated.
- Universal law
  4 $$\begin{aligned} y=f(r, \theta )= f(r,D,\eta _1,\zeta _1,\eta _2,\zeta _2) = D(w+ \eta _1)^{-\zeta _1}(1 - w + \eta _2)^{\zeta _2} \end{aligned}$$

where *y* is the size, $D,\eta _1, \eta _2,\zeta _1, \zeta _2$ are positive parameters to be calibrated and:

$$\begin{aligned} w = \frac{r - 1}{N - 1}, \end{aligned}$$

where *r* is the rank and *N* is the maximum rank – which coincides with the number of the ranked data.

For choosing among the aforementioned alternative rank-size curves, we evaluate their performances in terms of goodness of fit over the considered networks and for each centrality measure. Specifically, we estimate the parameters for each rank-size curve, by solving the following least-squares minimization problem:

5 $$\begin{aligned} \min _{\theta } \left[ y - {\hat{f}}(r, \theta )\right] ^2 \end{aligned}$$

where ${\hat{f}}(r,\theta )$ is the size predicted by the rank *r* according to the specific rank-size curve $f\left( r, \theta \right)$ and $\theta$ is the vector collecting the rank-size parameters. To estimate the parameters, we adopt the Levenberg-Marquardt algorithm (Levenberg, 1944; Marquardt, 1963). For measuring the goodness of fit we compute the $R^2$ associated with each rank-size curve by selecting the rank-size curve with the highest value of $R^2$ (see e.g., (Ausloos & Cerqueti, 2016; Cerqueti & Ficcadenti, 2022; Ficcadenti et al., 2022)).

Since we evaluate the rank-size curves associated with the networks over different years, one can hypothetically experience different best fitting curves over time for a fixed centrality measure. If this is the case, we select the rank-size curve with the highest average $R^2$ over the years. However, as we will see in the empirical experiments, we can substantially identify a rank-size curve that is the best one for all the considered years.

### Clustering procedures

The proposed rank-size clustering approach considers the difference in parameters estimated by a rank-size law as the dissimilarity among the statistical units, i.e. the complex networks. Let us collect the rank-size law parameters in the following matrix:

6 $$\begin{aligned} {\varvec{\Theta }} = \begin{bmatrix} \theta _{1,1} &{} \dots &{} \theta _{1,k} &{} \dots &{} \theta _{1,K}\\ \vdots &{} \dots &{} \vdots &{} \dots &{} \vdots \\ \theta _{i,1} &{} \dots &{} \theta _{i,k} &{} \dots &{} \theta _{i,K}\\ \vdots &{} \dots &{} \vdots &{} \dots &{} \vdots \\ \theta _{N,1} &{} \dots &{} \theta _{N,k} &{} \dots &{} \theta _{N,K} \end{bmatrix} \end{aligned}$$

of dimension $N \times K$, where *K* is the number of rank-size law parameters and *N* the number of networks, i.e. the years ($N=14$ in our case). For example, in the case of power law in (1), the matrix (6) can be written as follows:

7 $$\begin{aligned} {\varvec{\Theta }} = \begin{bmatrix} A_{1} &{} \alpha _1\\ \vdots &{} \vdots \\ A_{i} &{} \alpha _i\\ \vdots &{} \vdots \\ A_{N} &{} \alpha _N\\ \end{bmatrix} \end{aligned}$$

In this paper, we propose to cluster networks on the basis of their rank-size relationships. Thus, let us define $\Theta _i = \left( \theta _{i,1}, \theta _{i,2}, \dots , \theta _{i,K} \right)$ the vector containing the rank-size parameters for a given network *i* $(i=1,\dots , N)$. $\Theta _i$ is the *i*-th row of matrix ${\varvec{\Theta }}$ in (6). We compute the dissimilarity between two networks *i* and *j* ($i=1,\dots , N; j=1,\dots ,N$) by means of the following rank-size dissimilarity:

8 $$\begin{aligned} D_{i,j} = \sqrt{\left( \Theta _i - \Theta _j \right) ' \left( \Theta _i - \Theta _j\right) } = \sqrt{\sum _{k=1}^{K} \left( \theta _{i,k} - \theta _{j,k} \right) ^2} \end{aligned}$$

For clustering networks, we consider the fuzzy *k*-medoids algorithm (Krishnapuram et al. , 2001) that is based on the solution of the following problem:

9 $$\begin{aligned} \min :&\quad \sum _{i=1}^{N} \sum _{c=1}^{C} u_{i,c}^m D_{i,c}^2 \\&\text {s.t.} \sum _{i=1}^{N} \sum _{c=1}^{C} u_{i,c} = 1 \nonumber \end{aligned}$$

where $u_{i,c}$ is the membership degree of the *i*-th object to the *c*-th cluster, *m* is the fuzziness parameter and $D_{i,c}^2$ is the squared distance (8) between the *i*-th object with the *c*-th cluster centroid. The membership degree indicates the degree to which an *i*-th statistical unit – an *i*-th network in our case – belongs to *c*-th cluster. While in fuzzy clustering each *i*-th network belongs to all the *C* clusters with a given membership degree $u_{i,c}$, with a not fuzzy clustering approach each *i*-th unit is assigned to *c*-th cluster with the highest membership, that is $u_{i,c}$ is binary, either 0 (the *i*-th network does not belong to the *c*-th cluster) or 1 (the *i*-th network belongs to the *c*-th cluster).

Two important choices are the selection of the fuzziness parameter *m* and the number of clusters *C*.

The fuzziness parameter has to be chosen within the interval $m \in (1, +\infty )$, avoiding very large values. Indeed, for large values of *m* we get a very fuzzy partition, where all the statistical units have memberships equal to 1/*C* to each *c*-th cluster (D’Urso, 2015). Some authors (see e.g., Choe & Jordan , 1992) show that the performance of fuzzy clustering algorithms is not so sensitive to the variation of the fuzziness parameter, particularly for relatively small values of *m*. Therefore we choose $m=1.5$, also in line with previous studies (see e.g., Krishnapuram et al. , 2001).

In order to choose the number of clusters *C*, we consider the value of *C* maximizing the Fuzzy Silhouette (FS) criterion of Campello and Hruschka (2006). The FS introduces fuzziness in the Average Silhouette Width (ASW), which is a well-established validity index for evaluating the quality of a partition  (Arbelaitz et al., 2013; Batool & Hennig, 2021), which measures the within-cluster cohesion and inter-cluster dispersion. The Silhouette for an *i*-th object can be computed as follows:

10 $$\begin{aligned} S_{i}=\frac{(b_{i}-a_{i})}{\max \{b_{i},a_{i}\}} \end{aligned}$$

where $a_i$ is the average distance of the *i*-th units to the other units belonging to the same cluster *c* and $b_i$ is the average distance of the same unit to others belonging to the closest different cluster $c^{\prime }\ne c$ – we use the Euclidean distance (8) in our case. In other words, for an *i*-th unit belonging to a cluster *c* we have that(Batool & Hennig, 2021):

$$\begin{aligned} a_i=\frac{1}{n_c-1} \sum _{j=1}^{n_c-1} D_{i,j} \quad \text{ and } \quad b_i=\min _{c^{\prime } \ne c} \frac{1}{n_{c^{\prime }}} \sum _{j=1}^{n_{c^{\prime }}} D_{i,j} \end{aligned}$$

where $n_c$ denotes the size of cluster *c* and $n_{c^{\prime }}$ the size of cluster $c^{\prime } \ne c$. The quantity $\sum _{j=1}^{n_c-1} D_{i,j}$ is the sum of the distances – computed according to (8) – between the *i*-th unit and all the remaining $n_c -1$ units belonging to the same cluster *c*. Therefore, $a_i$ is the average distance of the *i*-th network of research projects to the other networks belonging to the same cluster *c*. Then, the term $\sum _{j=1}^{n_{c^{\prime }}} D_{i,j}$ is the sum of the distances between the *i*-th unit from the $n_{c^{\prime }}$ belonging to the $c^{\prime }$ cluster. Hence, $b_i$ is the average distance of the *i*-th network to others belonging to the closest different cluster $c^{\prime }\ne c$, as we take the minimum among the sums over the $C-1$ clusters different from *c*.

A large Silhouette value $S_i$ means that the *i*-th unit is closer to those belonging to its cluster than to the others belonging to the closest different cluster. The ASW is computed by averaging the values of $S_i$ for all the *N* $(i=1,\dots ,N)$ units as follows:

$$\begin{aligned} \text {ASW}= \frac{1}{N} \sum _{i=1}^{N} S_i \end{aligned}$$

Therefore, the higher the ASW, the better the partition’s quality. The FS considers a weighted average for the Silhouettes $S_i$ instead of a simple average by using the membership degrees $u_{i,c}$ as weights, as follows:

11 $$\begin{aligned} FS=\frac{\sum _{i=1}^{N}(u_{i,c}-u_{i,c'})^\varepsilon S_{i}}{\sum _{i=1}^{N}(u_{i,c}-u_{i,c'})^\varepsilon } \end{aligned}$$

where $S_i$ is the Silhouette computed as in (10), $u_{i,c}$ and $u_{i,c'}$ are the first and second-largest elements of the *i*-th row of the fuzzy partition matrix, respectively. The parameter $\varepsilon \ge 0$ is a weighting coefficient that is usually set equal to 1. Therefore, the FS stresses the importance of units closely placed to the cluster prototypes in the case of high membership while reducing the importance of the units placed in overlapping areas for low membership values.

## Results and discussion

**Fig. 1:**
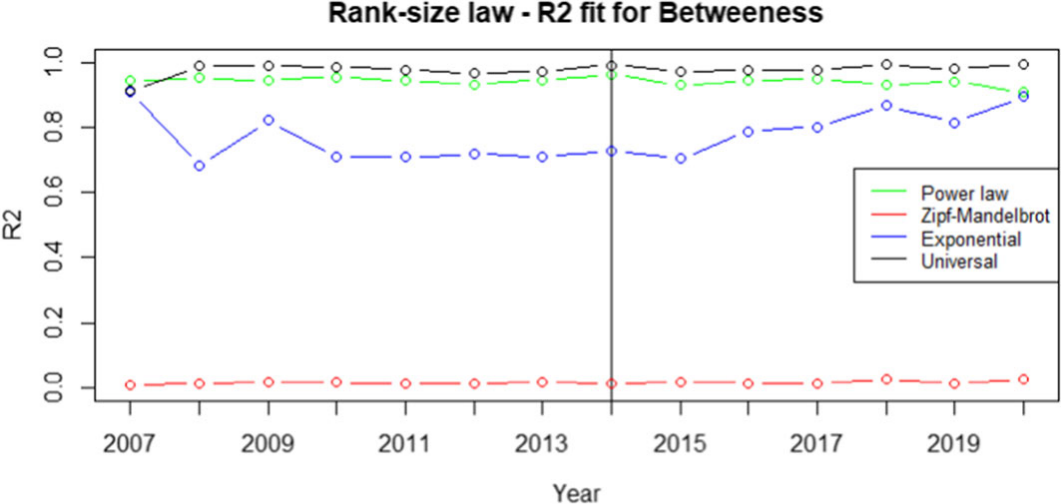
$R^2$ for betweenness centrality measure. The vertical line refers to the year 2014 and separates FP7 (left) from H2020 (right) projects. $R^2$ is defined as the ratio between the variance of the power law predictions and the variance of the actual ranked data. Therefore, the higher the value better is the power law fit with the actual ranked data. Higher the value, the better the fit. We choose the rank-size law with the highest $R^2$ value

**Fig. 2:**
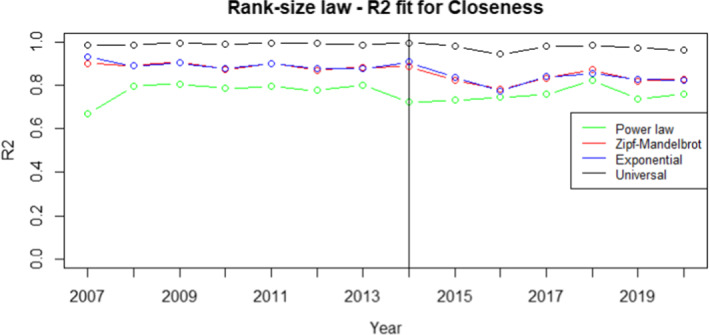
$R^2$ for closeness centrality measure. The vertical line refers to the year 2014 and separates FP7 (left) from H2020 (right) projects. $R^2$ is defined as the ratio between the variance of the power law predictions and the variance of the actual ranked data. Therefore, the higher the value better is the power law fit with the actual ranked data. Higher the value, the better the fit. We choose the rank-size law with the highest $R^2$ value

**Fig. 3:**
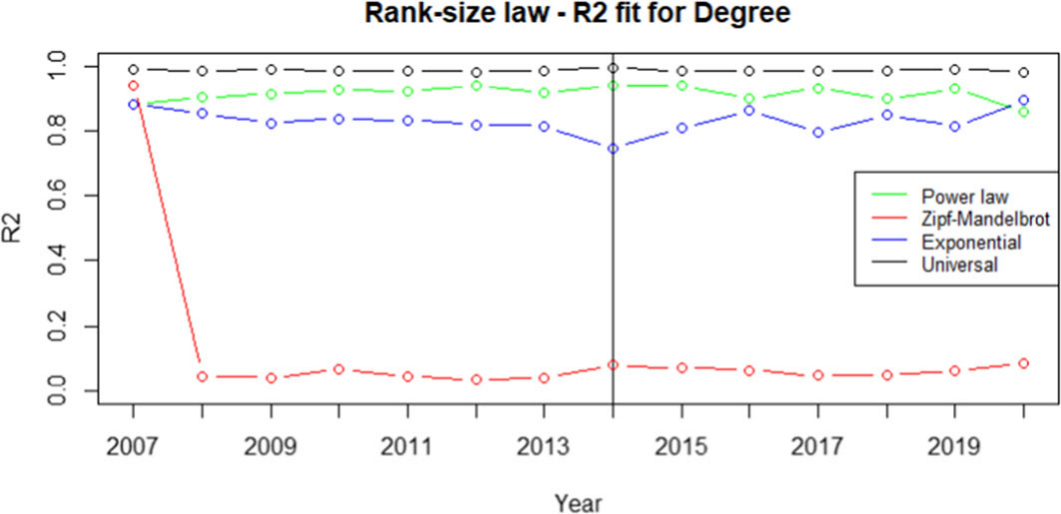
$R^2$ for degree centrality measure. The vertical line refers to the year 2014 and separates FP7 (left) from H2020 (right) projects. $R^2$ is defined as the ratio between the variance of the power law predictions and the variance of the actual ranked data. Therefore, the higher the value better is the power law fit with the actual ranked data. Higher the value, the better the fit. We choose the rank-size law with the highest $R^2$ value

**Fig. 4:**
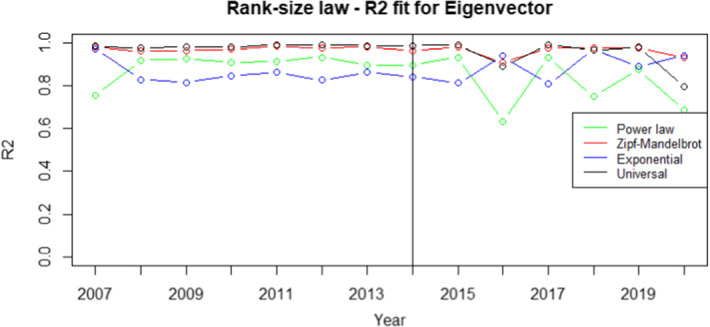
$R^2$ for eigenvector centrality measure. Vertical line refers to year 2014 and separates FP7 (left) from H2020 (right) projects. $R^2$ is defined as the ratio between the variance of the power law predictions and the variance of the actual ranked data. Therefore, higher the value better is the power law fit with the actual ranked data. Higher the value, better the fit. We choose the rank-size law with the highest $R^2$ value

Our procedure starts by estimating the rank-size laws for each of the centrality measures discussed in Table 6. We choose the rank-size law that better fits the data. Particularly, we aim to find a unique law valid for modelling all the networks (i.e. the years) for each considered centrality measure. We compare the rank-size laws discussed in Section 3.2 in terms of $R^2$, which is defined as the ratio between the variance of the power law predictions and the variance of the actual ranked data. Therefore, the higher the value better is the power law fit with the actual ranked data. In particular, we choose the rank-size law whose fit is better than others for all the 14 yearly networks. The values of the $R^2$ obtained with the universal law are excellent and very close to the maximum value of 1 (see Figs.  1, 2, 3, 4).

From the analysis of Figs. 1, 2, 3, 4, it is evident that the universal law fits better the data for most of the centrality measures, since its lines (the black ones) are above the alternatives for all the considered years. Only in the case of eigenvector centrality (see Fig. 4) the line corresponding to the $R^2$ of the universal law in (4) is lower than the power law in two years (2016 and 2020). However, it can be observed that the power law is very close to the universal law in terms of goodness of fit for these years, so there is no need of selecting a different best-fit law. Additional evidence supporting this conclusion is provided in Table 7, showing the average $R^2$ over the years for each rank-size law according to the alternative centrality measures.

**Table 7:** Average $R^2$ for each rank-size law

	Power law	ZM law	Exp law	Universal law
Betweenness	0.9428	0.0155	0.7763	0.9784
Closeness	0.7652	0.8627	0.8658	0.9835
Degree	0.9157	0.1193	0.8316	0.9883
Eigenvector	0.8545	0.9649	0.8733	0.9674

On the rows we have the considered centrality measures, while on the columns we have the families of rank-size curves: Power law is the one in (1), ZM law is the Zipf-Mandelbrot law in (2), Exp law is the Exponential law in (3) and Universal law is the (4)

**Fig. 5:**
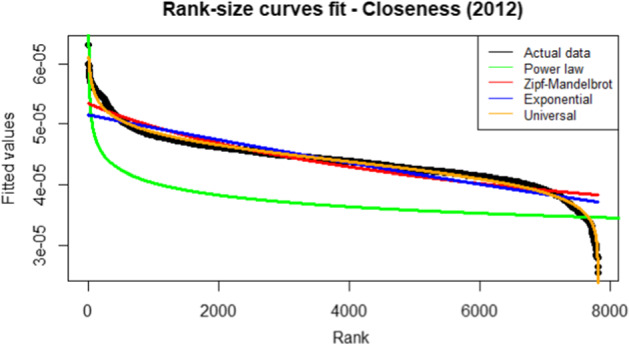
Fitted versus actual values - closeness centrality measure for the network in the year 2012. Ranked actual data refers to the network’s nodes, which are ranked in terms of a given centrality measure. Fitted values refer to the predictions obtained with alternative rank-size laws. The rank-size curve whose predictions overlap most with the actual ranked data is the one with the best fit

Similar conclusions can be derived by comparing the rank-size curve with actual data. Actual data refers to the network’s nodes, which are ranked in terms of a given centrality measure. For example, Fig. 5 shows the comparison considering the network in the year 2012, whose nodes are ranked according to the closeness centrality measure.

**Fig. 6:**
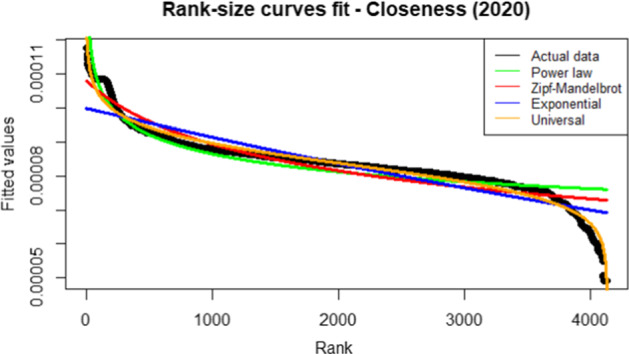
Fitted versus actual values - closeness centrality measure for the network in the year 2020. Ranked actual data refers to the network’s nodes, which are ranked in terms of a given centrality measure. Fitted values refer to the predictions obtained with alternative rank-size laws. The rank-size curve whose predictions overlap most with the actual ranked data is the one with the best fit

From Fig. 5 it is evident the superior fit obtained with the universal law. The same results are obtained in Fig. 6 that shows the comparison for the year 2020.

The arguments above justify the use of the universal law in (4) for clustering the networks.

Therefore, we have that $K=5$ and $\theta _{\cdot , 1}=D_{\cdot }$, $\theta _{\cdot , 2}=\eta _{1,\cdot }$, $\theta _{\cdot , 3}=\zeta _{1,\cdot }$, $\theta _{\cdot , 4}=\eta _{2,\cdot }$, $\theta _{\cdot , 5}=\zeta _{2,\cdot }$, to be computed over the considered networks.

The estimated rank-size parameters of each centrality measure are shown in Table 8.

Each panel of Table 8 contains a matrix ${\varvec{\Theta }}$ as shown in (6) for any given centrality measure.

The panels in Table 8 represent the entries of the clustering exercise for the four centrality measures.

**Fig. 7:**
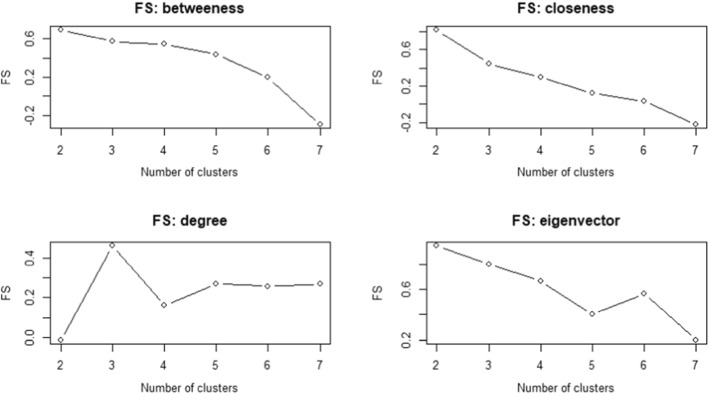
Fuzzy Silhouette for different number of clusters and alternative centrality measures. We choose the number of clusters (x-axis) that maximizes the Fuzzy Silhouette (y-axis)

A crucial preliminary step is the selection of the number of clusters *C*. To this aim, we compute the FS described in Subsect. 3.3. The values of the FS are shown in Fig. 7.

Accordingly, we choose $C=2$ clusters for betweenness, closeness and eigenvector centrality measures, while $C=3$ for the degree measure. The results of the rank-size clustering, in terms of crisp assignment, are shown in Table 9. With crisp assignment we adopt a binary approach to the assignment, meaning that each statistical unit – a network in our case – is assigned to the cluster with the highest membership degree.

**Table 8:** Estimated rank-size curve coefficients of the Universal law (4). Different panels refer to alternative centrality measures

Years	D	$\eta _1$	$\zeta _1$	$\eta _2$	$\zeta _2$
Panel A: betweenness centrality					
2007	0.1257	0.1643	6.5038	0.0000	2.2603
2008	23.5449	0.0000	0.5385	0.5373	14.6620
2009	37.0482	0.0002	0.5537	0.2970	22.4469
2010	23.9427	0.0000	0.5506	0.5191	14.9906
2011	26.0452	0.0000	0.5463	0.5269	14.7775
2012	22.6805	0.0000	0.5506	0.5606	14.6665
2013	21.6792	0.0000	0.5749	0.6069	13.5803
2014	21.6877	0.0001	0.5449	0.4675	15.5515
2015	25.2991	0.0000	0.5243	0.5207	15.7597
2016	26.6434	0.0001	0.5642	0.4932	15.6801
2017	22.8072	0.0001	0.6249	0.5699	13.6114
2018	40.6537	0.0003	0.6773	0.3619	17.1845
2019	33.4681	0.0001	0.5804	0.4058	17.6861
2020	41.1999	0.0002	0.4072	0.2308	29.0501
Panel B: closeness centrality					
2007	0.0008	0.0001	0.0315	0.0298	0.1859
2008	0.0000	0.0037	0.0574	0.0003	0.0616
2009	0.0001	0.0034	0.0559	0.0000	0.0587
2010	0.0000	0.0017	0.0507	0.0000	0.0612
2011	0.0000	0.0023	0.0502	0.0001	0.0588
2012	0.0000	0.0003	0.0426	0.0000	0.0598
2013	0.0000	0.0030	0.0561	0.0000	0.0574
2014	0.0001	0.0014	0.0456	0.0001	0.0873
2015	0.0000	0.0002	0.0400	0.0000	0.0680
2016	0.0000	0.0004	0.0484	0.0000	0.0619
2017	0.0000	0.0002	0.0422	0.0000	0.0620
2018	0.0000	0.0011	0.0529	0.0000	0.0512
2019	0.0000	0.0003	0.0426	0.0000	0.0676
2020	0.0001	0.0020	0.0576	0.0001	0.0714
Panel C: degree centrality					
2007	21.2182	0.0011	0.2533	0.1459	1.0085
2008	0.1902	0.0006	0.5151	1.4824	5.6010
2009	0.2421	0.0007	0.5220	1.7825	4.5466
2010	0.1812	0.0003	0.5063	1.5554	5.3086
2011	0.2924	0.0006	0.5550	1.7997	4.2379
2012	0.3208	0.0003	0.5493	1.8622	3.8800
2013	0.3105	0.0003	0.4923	1.2954	5.7294
2014	0.2006	0.0002	0.4215	1.7583	4.9107
2015	0.3654	0.0003	0.5189	1.7546	4.0994
2016	0.2199	0.0010	0.5873	1.9640	4.2512
2017	0.3747	0.0004	0.5242	1.9347	3.8781
2018	0.1732	0.0006	0.4935	1.6134	5.5190
2019	0.2708	0.0005	0.5325	2.0212	4.1447
2020	0.0437	0.0024	0.5247	2.1504	5.6003
Panel D: eigenvector centrality					
2007	0.0476	0.0281	0.4333	0.3856	4.6012
2008	0.0067	0.0006	0.6479	0.0000	1.2692
2009	0.0084	0.0005	0.6080	0.0000	1.2876
2010	0.0069	0.0008	0.6742	0.0000	1.2995
2011	0.0003	0.0007	0.6422	0.8946	4.9160
2012	0.0004	0.0003	0.5704	0.8640	5.0126
2013	0.0057	0.0010	0.7274	0.0000	1.4102
2014	0.0008	0.0006	0.4986	0.8217	5.6913
2015	0.0060	0.0004	0.6474	0.0000	1.6065
2016	0.0000	0.0034	2.0328	0.1602	1.3528
2017	0.0004	0.0003	0.5797	0.9997	4.5830
2018	0.0036	0.0065	1.0833	0.0000	1.9099
2019	0.0004	0.0014	0.7043	1.0984	4.1965
2020	0.0412	0.0003	0.4584	0.0000	1.7807

**Table 9:** Rank-size clustering (fuzzy *k*-medoids)—crisp assignment. With crisp assignment we adopt a binary approach to the assignment, meaning that each statistical unit—a network in our case—is assigned to the cluster with the highest membership degree. The medoids are in bold

	Betweenness	Closeness	Degree	Eigenvector
2007	1	2	3	1
2008	1	1	2	2
2009	**2**	1	1	2
2010	1	1	**2**	2
2011	1	1	1	**1**
2012	1	1	3	1
2013	**1**	1	2	**2**
2014	1	**2**	2	1
2015	1	1	**1**	2
2016	1	**1**	1	2
2017	1	1	**3**	1
2018	2	1	2	2
2019	2	1	1	1
2020	2	1	2	2

The clusters obtained with degree (three clusters) and eigenvector (two clusters) centrality measures are the most balanced ones. Indeed, with the degree, we identify three clusters of size 5 (cluster 1), 6 (cluster 2) and 3 (cluster 3), while with the eigenvector measure, we have two clusters of almost equal size. On the other side, betweenness identifies two groups, with the first one (cluster 1, 10 units) being more numerous than the other one (cluster 2, 4 units). In comparison, closeness also identifies two groups but with one (cluster 2) including only two networks (i.e. 2007 and 2014). The medoids (highlighted in bold) are very different and change on the basis of the considered centrality measure. In terms of FS, the partition obtained with the eigenvector has the highest values (FS equal to 0.9479), meaning that the groups are compact and well separated. However, in the other cases, we still have good results, with rather large FS values. In particular, we have an FS equal to 0.6935 for betweenness and 0.8142 for closeness, so the obtained partitions are quite satisfactory. We obtain the lowest silhouette with the degree centrality measure, but with a value almost equal to 0.5.

The membership degrees are shown in Table 10.

**Table 10:** Rank-size clustering (fuzzy *k*-medoids): membership degrees associated to the assigned cluster. The membership degree indicates the degree to which an *i*-th network belongs to *c*-th cluster

	Betweenness	Closeness	Degree	Eigenvector
2007	0.89	0.70	0.34	1.00
2008	1.00	0.99	1.00	1.00
2009	1.00	0.99	0.76	1.00
2010	1.00	1.00	1.00	1.00
2011	0.99	1.00	0.97	1.00
2012	1.00	1.00	0.98	1.00
2013	1.00	0.99	0.99	1.00
2014	1.00	1.00	0.89	1.00
2015	0.99	0.94	1.00	1.00
2016	0.97	1.00	0.76	0.99
2017	1.00	1.00	1.00	1.00
2018	0.99	0.99	1.00	1.00
2019	0.95	0.97	0.54	1.00
2020	0.99	0.84	0.95	1.00

Overall most of the networks are assigned to the clusters with high membership degrees. The only fuzzy units – for the definition of a unit as “fuzzy”, we can follow the indications of previous studies (Dembele & Kastner, 2003; D’Urso & Maharaj, 2009; Maharaj et al., 2010) suggesting thresholds of 0.7 for $C=2$ clusters and 0.6 for $C=3$ – are the network of the European research project in the years 2007 and 2019 (degree centrality measure, third column of Table 10) since 2007 has a membership of around 0.3 and 2019 a value that is slightly larger than 0.5.

The rank-size curve parameters associated with the clusters’ medoids are in Table 8 in light of the results of Table 9. However, to better insight the clusters’ whole composition, we analyze the average parameters’ value within each cluster instead of looking at the single medoids. Table 11 and Table 12 show the mean and standard deviation of the Universal law parameters associated with the networks – constructed in terms of the different centrality measures – included in the clusters. Hence, Table 11 and Table 12 provide details about the main characteristics of the networks included in the clusters.

**Table 11:** Average Universal rank-size curve parameters $(D,\eta _1, \zeta _1, \eta _2, \zeta _2)$ within each cluster, obtained with alternative centrality measures (the Panels)

Cluster	Average				
	D	$\eta _1$	$\zeta _1$	$\eta _2$	$\zeta _2$
Panel A: betweenness					
1	21.445558	0.016476	1.152306	0.480214	13.553996
2	38.092450	0.000193	0.554654	0.323845	21.591901
Panel B: closeness					
1	0.000044	0.001537	0.049730	0.000057	0.061649
2	0.000446	0.000749	0.038547	0.014925	0.136600
Panel C: degree					
1	0.278133	0.000602	0.543145	1.864427	4.255939
2	0.183231	0.000723	0.492226	1.642571	5.444838
3	7.304575	0.000616	0.442300	1.314278	2.922228
Panel D: eigenvector					
1	0.008333	0.005244	0.571429	0.843994	4.833430
2	0.009810	0.001692	0.859906	0.020030	1.489544

**Table 12:** Standard deviation of Universal rank-size curve parameters $(D,\eta _1, \zeta _1, \eta _2, \zeta _2)$ within each cluster, obtained with alternative centrality measures (the Panels)

Cluster	St. Dev.				
	D	$\eta _1$	$\zeta _1$	$\eta _2$	$\zeta _2$
Panel A: betweenness					
1	3.591233	0.000091	0.111754	0.076487	5.508648
2	7.686183	0.051936	1.880535	0.173228	4.040807
Panel B: closeness					
1	0.000013	0.001308	0.006505	0.000087	0.005368
2	0.000535	0.000978	0.010018	0.020983	0.069683
Panel C: degree					
1	0.056060	0.000289	0.028423	0.119856	0.174431
2	0.08501880	0.0008260401	0.03682455	0.2918793	0.2962651
3	12.049579	0.000427	0.164119	1.012468	1.657305
Panel D: eigenvector					
1	0.019250	0.011217	0.097070	0.245984	0.509349
2	0.012931	0.002170	0.506027	0.056625	0.246589

Some differences can be highlighted. For betweenness, we observe that the second cluster is characterized by an average *D* value much greater than the one in cluster 1. Moreover, the within-group variability of *D* is larger in the second group than in the first one. Next, cluster 2 shows values of both $\eta _1$ and $\zeta _1$ much smaller than those in cluster 1 (0.0001 versus 0.016 for $\eta _1$, while 0.55 versus 1.15 for $\zeta _1$) but with a higher degree of heterogeneity within the cluster for both the parameters. The value of $\zeta _2$ is larger in the second cluster than in the first one but with lower within-group variability. According to closeness, we find that the second group has an average *D* value greater than the one in cluster 1 but also $\eta _2$ e $\theta _2$ are larger, on average, for the second cluster. Overall, the second cluster shows a higher degree of variability for most of the parameters, suggesting the presence of a greater within-group heterogeneity. Considering the degree centrality measure, we identify the presence of a group (cluster 3) with an average value of *D* that is exceptionally larger than the other two groups (7.3 versus 0.28 e 0.18). This happens since cluster 3 includes the network in 2007, which is characterized by a very large value of *D* with respect to the other years. Therefore, also the variability in terms of *D* is exceptionally high for this cluster compared with the other two. According to the other parameters, the clusters show relatively similar values with the only exception represented by $\zeta _2$ for which clusters 1 and 2 have larger values than the one in cluster 3. In particular, the cluster with the lowest value of *D* is associated with the highest $\zeta _2$. The first group is characterized by a higher degree of homogeneity in terms of parameters’ variability since all the parameters show the lowest standard deviations. In the end, we analyze the results in terms of eigenvector centrality. Also in this case, we observe a distinction across the groups in terms of average *D* values, with the second group showing the larger value. Further, $\eta _2$ and $\zeta _2$ are larger for cluster 1, while the $\zeta _1$ associated with the second cluster is larger than cluster 1. Finally, standard deviations suggest that cluster 2 is characterized by higher variability in the parameter $\zeta _1$, while cluster 1 shows higher variability for the other parameters.

We now provide the analytic interpretation of the parameters in (4). The parameter *D* increases as the absolute value of the element at rank 1 – i.e., the highest size of the sample – increases. The parameters $\eta _1$ and $\zeta _1$ refer to the low ranks. Specifically, the value of $\eta _1$ increases as the deviation between the sizes at two consecutive (small) ranks – e.g., size at $r=1$ and $r=2$; $r=2$ and $r=3$, etc. – increases. A large value of $\zeta _1$ amplifies such an increasing behavior. Indeed, when $\eta _1$ is large and/or $\zeta _1$ is large, then the best-fit curve is steeper at low ranks. The parameters $\eta _2$ and $\zeta _2$ behave in a similar way but for large values of *r*, at high ranks. Therefore, large values of $\eta _2$ and $\zeta _2$ explain large values of the discrepancy between the sizes of two consecutive high ranks. The curve flattens at high ranks for low values of $\eta _2$ and/or $\zeta _2$.

**Fig. 8:**
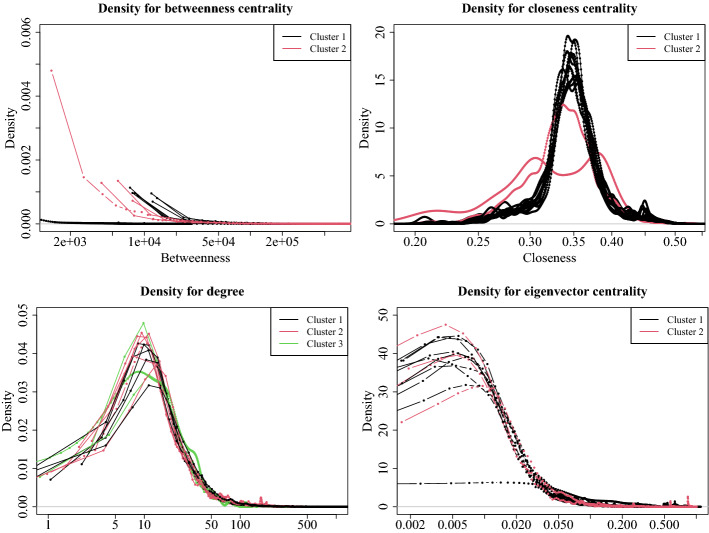
Density plots for the four centrality measures (top left: betweenness centrality; top right: closeness centrality; bottom left: degree centrality; bottom right: eigenvector centrality). Each plot reports the 14 curves for each network, colored according to their cluster membership

The obtained results can be then interpreted in the context of complex networks, with specific reference to the considered centrality measures. Figure 8 reports the density plots for such measures, i.e. betweenness, closeness, degree, and eigenvector centrality. Each plot reports the density plots for all the 14 networks colored according to their membership cluster.

We can observe a clear distinction between the two clusters for betweenness and closeness centrality measures. Indeed, for the first measure, the lowest values for networks in cluster 1 are considerably higher than the lowest values for networks in cluster 2. Differently, for the second measure, networks in cluster 1 have values around a common value, while networks in cluster 2 have very different behaviour (red curves) that are in accord with the results in Table 11 (Panel A and B) where the *D* and the other coefficients are different within each Panel.

In terms of social network analysis, we can observe from Table 9 that according to the betweenness, the networks are assigned from 2007 to 2017 to cluster 1 (except for 2009), highlighting the trend that goes from less to more scattered values. This means that intermediation tends to concentrate in hubs which are all public institutions (see Table 3). Regarding the closeness centrality, in cluster 2 we have the year 2007, which is the starting year, and 2014, with a density plot not so different from the others, i.e., information flows among the participants similarly along the time horizon.

**Fig. 9:**
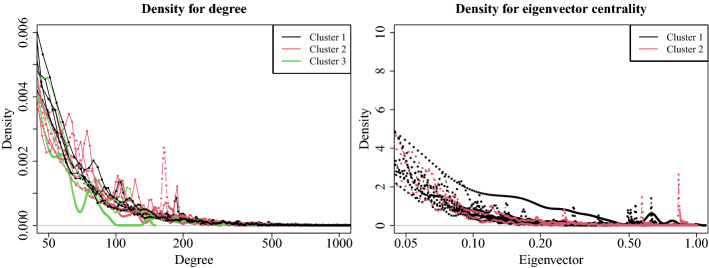
Density plots for the degree (left) and eigenvector centrality (right) measures limited to their highest values. Each plot reports the 14 curves for each network, colored according to their cluster membership

When we consider degree and eigenvector centrality measures, the difference between the clusters is less evident. In Fig. 9 we reported their density plots limited to values higher than a certain threshold, in particular, $k_i \ge 50$ for the degree centrality and $C_E \ge 0.05$ for eigenvector centrality.

The two plots let to observe the tails of the density plots in Fig. 8. We can note that for the degree, networks in cluster 2 are basically more variable than the networks in the other clusters (as confirmed by the values of the standard deviations in Table 11, Panel C). In contrast, the networks in cluster 3 tend to zero faster than those in the other cluster. In other words, maximum degrees are lower. Finally, networks in cluster 1 show more regular trends, with a slope to zero slower than that of the other networks.

Regarding the eigenvector centrality, values for networks in cluster 1 decrease with a smoothed slope than those in cluster 2, highlighting that networks in cluster 1 have more influential nodes than those in cluster 2.

The analysis performed in this section can be considered as an ex-post advanced evaluation of several years of the funded project in EU. Under this perspective, it could be of great help for practitioners and policymakers to understand under a different point of view if endogenous or exogenous factors have had an impact during the years of the FP7 and H2020 initiatives on the system as a whole.

In the case of the closeness, the starting years of the two initiatives are gathered in cluster 2, which clearly identifies the fact that in the first year of the two initiatives the system is less cohesive and it suffers a sort of cold start, having density and average path much higher than those in cluster 1. In other terms, since a short length of the shortest paths is usually considered a signal of self-organised systems, then cluster 2 identifies systems less self-organised.

## Conclusions

This paper addresses a central issue in the context of public policy evaluation: the analysis of scientific institutions in the context of research funding. In particular, we explore the organizations involved in European project funding, proposing an analysis of their interconnections based on joint research projects. We place ourselves within three methodological strands: first, the study of complex networks and their applications, which allows analyzing specific entities with their interconnections; second, the rank-size analysis, which allows studying the system resulting from disaggregated data that are properly ranked; third, the cluster analysis, which captures regularities and deviations among the considered statistical units. The application of such techniques to the specific context analyzed here enables us to derive relevant insights about the dynamics of the funded European research activity and the scientific institutions involved. The selection of a wide range of nodal centrality measures leads to a clear view of many aspects related to the research centers in Europe, hence identifying the relevance of institutions and years of research under different perspectives.

It is worth mentioning some main lines for future research. First, it is possible to complete the study of the considered research networks through other centrality measures by pointing specific attention to community detection and link formation. In this respect, the present work represents a crucial first step toward a deeper exploration of this challenging task. Second, one can implement different clustering strategies based on other concepts of distance with additional informative content. In so doing, it is possible to derive the regularities among research institutions and years of European research funds when taking different similarity criteria.

Finally, and importantly, we notice that while closeness is a measure affected by endogenous characteristics, betweenness, degree and eigenvector centrality are more related to the qualities of the nodes or to external factors. For the specific case of participants to FP7 and H2020, their actions, commitment and perceptions could be strongly influenced by programs of incentives, macroeconomics factors, local policies as well as from innovation paths or market requirements. Under these perspectives, it is of paramount importance to study the relationships among networks clustering and the above mentioned factors – and potentially many others - and we will devote to these topics further researches in the next future.

## Footnotes

1 European Commission Research & Innovation, FP7 in brief: https://op.europa.eu/en/publication-detail/-/publication/56f1ac29-3315-4ca2-95de-b1e098838965; accessed: February 17th, 2022.

2 Horizon 2020 definitive reference documents: https://ec.europa.eu/programmes/horizon2020/en/official-documents; accessed: February 17th 2022.
